# Report of the Convalescent Homes Committee

**Published:** 1919-12-20

**Authors:** 


					REPORT OF THE CONVALESCENT HOMES COMMITTEE.
1. The President and General Council have this year
fixed the sum available for distribution amongst con-
valescent homes and consumption sanatoria at ?12,000, as
flgainst ?10,000 in 1918. This increased amount has
assisted the Committee to meet the increased demands on
tho Fund, due to the reopening of convalescent homes
closed during the war and 'to the unavoidable rise in the
cost of maintenance of beds at sanatoria, referred to in
l?ter paragraphs of this report.
2. The number of applications eligible for consideration
amounted to forty-nine from convalescent homes and nine
from consumption sanatoria, as against forty-five and nine
respectively last year.
3. In dealing with all applications, whether from sana-
toria or from convalescent homes, the Committee have, as
usual, taken into consideration the question of comparative
urgency of need, as shown by the audited accounts and
balance sheets of the various institutions to December 31
last. In the case of consumption sanatoria they have also
taken note of the proportion of the total work of each
institution which is covered by the receipts from public
authorities. As these and other circumstances affecting- the
relative claims of the different institutions vary fror^i year
to year, it must not be assumed that a reduction in tho
grant to any particular institution, whether for mainten-
ance or for capital purposes, or even the absence of a
grant, implies dissatisfaction.
4. Most of the convalescent homes which ceased, on
account of the war, to be occupied by convalescent patients
from London have been reopened during the course cf
1919. The amount allotted to convalescent homes has
therefore been increased, though not as much as would
have been required had all these homes been doing their
normal work for the whole of the year.
5. The amount distributed to consumption sanatoria is
also greater than last year, owing to the rise in the cost
of maintaining beds and to the fact that the number cf
beds reserved, by the Fund for the use of patients in Lon-
don hospitals has been increased by two. The accommo-
dation secured this year amounts to sixty-six beds at six
966 THE HOSPITAL Decembee -20, 1919.
King Edward's Hospital Fund?(continued).
sanatoria, situated as follows: Ten beds for children at
tho Children's ^Sanato.rium, Holt, Norfolk; four beds at
the Daneswood Sanatorium (Jewish), Woburn Sands, Beds;
ten be<ls at the Devon and Cornwall Sanatorium, South
Brent, Devon; six beds at the Ke}ling Sanatorium, Holt,
Norfolk; twenty-four beds at tho Sanatorium of the
National Association for the Establishment and Mainten-
ance of Sanatoria for Workers, Benenden, Kent; twelve
beds at tho Northamptonshire Sanatorium, Creaton,
Northants.
6. The Committee recommend that the beds thus reserved
be allocated amongst London hospitals as follows: Chest
hospitals: Thirteen beds to the City of London Hospital
for Diseases of the Chest; six beds to the Royal Hospital
for Diseases of the Chest. General hospitals: Two beds
to Charing Cross -Hospital; nine beds to Guy's Hospital;
ono bed to King's College Hospital; eleven beds to tho
London Hospital; five beds to the Middlesex Hospital; one
bed to the Royal Free Hospital; four beds to St. George's
Hospital; two beds to St. Mary's Hospital; six beds to
St. Thomas's Hospital; four beds to University College
Hospital; two beds to Westminster Hospital.
7. In view of the universal demand for facilities for
dealing with tuberculosis, the small number of men's beds
which the general hospitals appear to be able to use in
this way is a matter which may require further considera-
tion next year. On the other hand, the Committee have
been unable to secure enough women's beds to satisfy the
demand.
8. The inspection of consumption sanatoria in the country,
which had been suspended since 1916, owing to the restric-
tions on travelling facilities both by rail and by motor, was
resumed this year. The Committee .aro greatly indebted
to the visitors who volunteered for these distant journeys
under present conditions, and are recommending that
various extracts from their reports should be sent on with
the grants for the observations of the Committees of the
institutions concerned.
9. The grants recommended to the various institutions aro
appended.
For the Committee, William H. Bennett, Chairman.
November 25, 1919.

				

